# Recent updates on Sintilimab in solid tumor immunotherapy

**DOI:** 10.1186/s40364-020-00250-z

**Published:** 2020-12-01

**Authors:** Xuhong Liu, Yong Yi

**Affiliations:** 1 Department of Oncology, The Second Xiangya Hospital, Central South University, Changsha, 410011 Hunan China; 2 Liver Cancer Institute, Zhongshan Hospital and Shanghai Medical School, Key Laboratory for Carcinogenesis & Cancer Invasion, The Chinese Ministry of Education, Fudan University, Shanghai, 200032 China

**Keywords:** Solid tumors, Sintilimab, PD-1 inhibitor, Immunotherapy, Clinical Progress

## Abstract

In recent years, there have been advancements in traditional patterns of tumor therapy with the adoption of immunotherapy. Its application with or without other combined regimens has attracted attention from clinicians. Sintilimab (Tyvyt®), a highly selective fully human IgG4 monoclonal antibody, blocks the binding site of programmed cell death protein 1 (PD-1), thereby, inhibiting the interaction between PD-1 and its ligands (PD-L1/2) to restore the endogenous anti-tumor T cell responses. Sintilimab has been proven to be clinically beneficial in multiple solid tumor therapies. Combination therapy and monotherapy have shown potential and encouraging anti-tumor efficacy with controllable and acceptable toxicities. The combination therapy is more likely to be a novel and promising therapeutic option. This study provides an overview of the status of sintilimab-based clinical trials in various solid tumors.

## Introduction

Immune checkpoint proteins, including PD-1 and cytotoxic T-lymphocyte-associated antigen 4 (CTLA-4), initiate signaling pathways that suppress T-cell anti-tumor effect [1].

Following the binding of immune checkpoint inhibitors (ICIs) to immune checkpoint proteins on the surface of T cells, tumor-mediated immune suppression is reversed [2]. Currently, clinical application of ICIs are mainly focused on anti-CTLA4 antibodies (ipilimumab, tremelimumab), anti-PD-1 antibodies (pembrolizumab, nivolumab, cemiplimab) and anit-PD-L1 antibodies (atezolizumab, avelumab and durvalumab). Hence, ICIs have immensely revolutionized and transformed cancer therapeutic modes [1, 3].

Sintilimab (Innovent Biologics, Suzhou, China), a type of PD-1 antibodies originally co-developed by Innovent Biologics, Eli Lilly and Company, has been approved for clinical management of either relapsed or refractory classical Hodgkin’s lymphoma (RR-c-HL)after more than second-line systematic chemotherapy [4]. Notably, Sintilimab was included in the 2019 edition of The Lymphoma Diagnosis and Treatment Guide of The Chinese Society of Clinical Oncology [4].

## Preclinical efficacy of Sintilimab

Immune activation effects are maximized when immune checkpoint receptors are blocked and reach their saturation. This is mediated by both the concentration and the affinity of the antibody to the receptor. Despite anti-PD-1 antibodies (pembrolizumab (Keytruda, MK-3475) and nivolumab (Opdivo, MDX-1106)) exhibiting the highest affinity for human PD-1 and a lower off-rate, the PD-1 receptor occupancy of sintilimab on circulating T cells is superior, thus indicating a possibility of a continuous blockade of the PD-1-PD-L1/2 pathway [5] (Table 1). The drug occupies the binding site for a long time even after plasma concentrations have decreased, which may lengthen its effect [5].

**Table 1:** Affinity of Three Types of anti-PD-1 Antibodies [5]

	Sintilimab	Keytruda	Opdivo
AUC _inf_ (h· μg/mL)	7846.554	8048.861	9349.858
t _1/2_ (h)	35.623	42.453	43.505
C_max_ (ug/mL)	218.519	224.217	238.710
V_ss_ (mL)	1.262	1.527	1.299
CL (mL/h)	0.025	0.025	0.021
Mean PD-1 receptor occupancy	>95%	NR	>70%
Kd (1/s)	8.028E-5	4.090E+ 6	1.465E-3
K_a_ (1/Ms)	1.090E+ 6	4.090E+ 6	4.599E+ 5

*t*_*1/2*_ half-life, *NR* not reported, *K*_*d*_ dissociation constant, *V*_*ss*_ volume of distribution at steady state, *C*_*max*_ maximum observed concentration, *CL* clearance, *AUC*_*inf*_ area under the serum concentration − time curve extrapolated to infinity (AUC all + C last /λ z), *K*_*a*_ association constant

Previous studies established that the sustained average PD-1 receptor occupancy rates on circulating T cells in patients administered with MDX1106 (dose-independent) and sintilimab were > 70 and > 95%, respectively [6, 7]. Besides, the immunogenicity of the drug affects its clinical efficacy. Protein drugs induce humoral immune responses, leading to the production of anti-drug antibodies (ADAs) and neutralizing antibodies (NAbs) upon repeated injections [8]. This phenomenon has been closely associated with the reduction in efficacy and resistance to the antibody. In humanized mouse models, sintilimab was found to exhibit better anti-tumor effects than MDX-1106 and MK-3475 [5]. These findings demonstrate the potential of sintilimab as a novel, safe, effective anti-PD-1 antibody in the cancer immunotherapy regimens.

## Clinical Progress of Sintilimab in application

### Lymphoma

A clinical trial (NCT03114683, ORIENT-1) by Shi et al, aimed at determining the efficiency and safety of sintilimab among the Chinese population, involved intravenous administration of sintilimab (200 mg, Q3W) to RR-cHL patients. There were no fatal cases associated with the clinical trial [6]. Among patients with RR-cHL, the objective response rate (ORR) with sintilimab was 80.4%, whereas nivolumab and pembrolizumab each had 69% [5]. These outcomes positioned sintilimab as an original strategy for RR-cHL patients [6]. Regarding the results of Shi et al, Ansell SM reported identical findings.. He stated the similar durable 6-month progression-free survival rate of 77.6% for sintilimab was highlighted when compared with 77% for nivolumab and 69% for pembrolizumab [9]. Moreover, RR-cHL patients with HIV following the administration of sintilimab experienced no fatal irAEs [10].

Application of triplet therapy of sintilimab with decitabine and GDP (gemcitabine, DDP, and dexamethasone), enhanced clinical responses in a diffuse large B-cell lymphoma (DLBCL) patient with 17p deletion who was irresponsive to the 1st and the 2nd line treatments [11].

### Lung Cancer

#### Clinical trials

A trial of phase Ib in non-small lung cancer (NSCLC) was performed to evaluate the safety and efficacy of sintilimab in neoadjuvant therapy (registration number: ChiCTR-OIC-17013726). Two cycles of sintilimab (200 mg, 1st day and 22nd day) were administered to patients with resectable NSCLC at clinical-stage IA-IIIB followed by pulmonary operations after immunotherapy on the 43rd day. The patients achieved disease control rates (DCR), major pathologic response (MPR) and pathologic complete responses (pCR) of 90%, 40.5% and 16.2%, respectively. Toxicities from the administration were usually grade 1/2, indicating that patients with early-stage NSCLC could benefit from neoadjuvant immunotherapy and be well-tolerated [12]. Furthermore, it was established that a decrease in the SUV value of Positron Emission Tomography-Computed Tomography (PET-CT) may be used as a predictor for the pathological responses of NSCLC after sintilimab treatment [12].

In addition to neoadjuvant therapy, a combination of sintilimab and anlotinib (12 mg/d, 2 weeks on/1 week off) was investigated as a first-line choice for patients with NSCLC, which was a single-arm of phase I clinical trial (NCT03628521). According to the interim analysis, the ORR and disease control rate (DCR) was 77.3% and 100%, respectively. As for safety, 31.8% of 22 patients exhibited grade 3–4 treatment related adverse events (TRAE). The most frequent was hematuria. AEs were tolerable from the outcome, indicating an eminent anti-tumor effect due to the combination of anti-PD-1 with multi-target anti-angiogenesis drugs [13].

A randomized, double-blind, multi-center, phase III study (NCT03607539, ORIENT-11) was performed to compare sintilimab or placebo plus chemotherapy (pemetrexed and cisplatin/carboplatin) in treatment-naïve patients with locally advanced or metastatic non-squamous NSCLC and without sensitizing epidermal growth factor receptor (EGFR) or anaplastic lymphoma kinase (ALK) genomic aberration. After a median follow-up of 8.9 months, the median progression-free survival (mPFS) of the sintilimab group against the placebo group was 8.9 and 5.0 months (*p* < 0.00001), respectively. The PFS rates at 6 months was 68.3% for sintlimab-combination and 42.0% for placebo-combination. The PFS benefit in the sintilimab-combination group was observed in all PD-L1 subgroups. Although the median overall survival (mOS) data for the two groups were not met, it showed that the sintilimab group tended to precede the control at the 6th month. The ORR was 51.9% in the sintilimab-combination group and 29.8% in the placebo-combination group, respectively. The incidence of grade 3–4 AEs was 61.7% in the sintilimab-combination group and 58.8% in placebo-combination group while DCR was higher in the sintilimab-combination group (86.8%, 95% CI: 82.2–90.7) than in the placebo-combination group (75.6%, 95% CI, 67.3–82.7). With a manageable safety and tolerability profile in patients, a combination of sintilimab and chemotherapy exhibited a tremendous potential as a first-line regimen for non-squamous NSCLCs [14].

On the European Society for Medical Oncology (ESMO) congress 2020, ORIENT-12 (NCT03629925) was reported, a randomized, double-blind, phase III study to evaluate the safety and efficacy of sintilimab plus gemcitabine and platinum (GP) as a first-line option for patients with either locally advanced or metastatic squamous NSCLC. Regardless of PD-L1 expression (TPS ≥1% or < 1%), the combination was shown to benefit patients in mPFS (5.9 m vs 4.9 m, HR, 0.575; 95% CI 0.435–0.761; *P* = 0.00009), with an acceptable safety profile [15]. The OS data have not been reported yet.

A phase of Ib clinical trial (NCT02937116) was designed to assess the efficacy and safety of sintilimab-chemotherapy as a first-line therapeutic option  for both nsqNSCLC and sqNSCLC. In this study, 21 patients (pts) and 20 pts. were enrolled in nsqNSCLC group and sqNSCLC group, respectively. ORR was found to be 68.4% and 64.7%, respectively. mPFS was found to be 12.6 months and 6.5 months in the nsqNSCLC cohort and sqNSCLC cohort, respectively. Moreover, the mOS data were evaluated to be 18.9 months and 15.4 months in the nsqNSCLC group and sqNSCLC group, repectively.

It revealed that 90.5% of the participants suffered AEs in the nsqNSCLC cohort, with neutropenia being the most significant. The ratio of grade 3–4 AEs was 38.1% in the nsqNSCLC cohort, while in the sqNSCLC cohort, the AEs incidence was up to 100%, among which the most common was white blood cell (WBC) count decline. The proportion of grade 3–4 AEs was 85%. There were no fatal events associated with treatments in both two cohorts. The most frequent sintilimab-related AEs were ALT increase, most of which were grade 1–2. Comparatively, sintilimab-chemo therapy exhibited a higher incidence and severity of AEs in sqNSCLC than in nsqNSCLC.

Notably, the study revealed that PD-L1 expression, whether tumor proportion score (TPS) ≥ 1% or TPS < 1%, was not correlated with clinical response, and neither did the tumor mutation burden level (TMB). A high TCR Change (TCRC) clonality (> 1) was significantly associated with a better OS, while a low TCRC diversity (< 1) was closely related with a superior PFS [16].

Hitherto, a combination of sintilimab and chemotherapy has been used in advanced NSCLC. Unlike previous, studies that deemed PD-L1 expression to be a predictor for the evaluation of tumor efficacy post-immunotherapy [17], the predictive role of this parameter might be overturned upon sintilimab administration on patients with NSCLC. This outcome requires more large-scale clinical trials to be confirmed.

#### Case series

An elderly man diagnosed with stage IVb lung adenocarcinoma without the driver-gene mutation achieved a partial remission (PR) after being administered with a total of five cycles of sintilimab (Q3W, 200 mg) combined with pemetrexed plus carboplatin. The curative effect lasted for about 3 months after the discontinuation of sintilimab with chemotherapy due to cardiac and skin adverse events. However, the PFS was over 9 months [18], which coincided with the outcome of ORIENT-11 [14].

Not only the combination of sintilimab with GP but also the administration of sintilimab with nedaplatin plus paclitaxel on a stage IIIB squamous NSCLC(T3N2M0) patient showed an encouraging anti-tumor effect. After 3 cycles of treatments, the TPS of the patient dropped from 80% to 0% [19], demonstrating the potential therapeutic value of sintilimab in resectable NSCLC. In another study, a combination of sintilimab plus bevacizumab was administered on a stage IIIB lung adenocarcinoma patient with EGFR exon 19 del mutation to achieve a 6-month PFS, thus suggesting tolerance as well as safety [20].

Apart from NSCLC, sintilimab was used against small cell lung cancer (SCLC). The patient responded well because the sum of the diameter of the target lesions reduced by 12.23% following two cycles of sintilimab (3.5 mg/kg) along with docetaxel (1.5 mg/kg). However, due to paraneoplastic neurological syndromes (PNSs), sintilimab efficacies were terminated [21].

### Hepatocellular carcinoma

The regimen of sintilimab (200 mg, Q3W) and IBI305 (a bevacizumab biosimilar, 15 mg/kg, Q3W) was once used to manage on a patient with HBV-associated HCC multiple pulmonary metastases, low microsatellite instability (MSI-L), high PD-L1 expression and TMB-L. In this patient, there was a complete remission (CR) after 2 cycles of administration and the level of alpha-fetoprotein (AFP) decreased from 1181.46 ng/ml to normal. The liver function of the patient remained Child-Pugh A throughout [22].

A combination of atezolizumab and bevacizumab was approved as a first-line therapeutic choice for advanced or metastatic HCC [23]. Recently, a phase II clinical trial of sintilimab (200 mg IV Q3W) combined with IBI305 (15 mg/kg IV Q3W) as a first-line treatment option for advanced HCC (NCT03794440, ORIENT-32) was performed by Fan et al. They showed that mPFS reached 8.4 months (95% CI, 5.6, not reached) but not OS. DCR was a preeminent 83.3%, while ORR was 25%, with an acceptable safety profile. Since a total of 24 patients were enrolled in the phase II clinical study, a large patient population is recommended to ascertain our findings. However, a phase III clinical trial of this study to compare the efficacy of sintilimab plus IBI305 and sorafenib is ongoing [24].

### Gastric Cancer

A phase Ib clinical trial (NCT02937116) of sintilimab combined with oxaliplatin/capecitabine (CapeOx) as the first-line therapeutic option was performed for patients with locally advanced or metastatic gastric (G)/gastroesophageal junction (GEJ) adenocarcinoma without previous systemic treatment. The enrolled patients had more than 4 cycles of treatment with the median treatment duration being 6.2 months (range 2.1–10.4). Grade 3–4 TRAEs occurred in 55.0% out of 20 patients, in which the most frequent TRAE was a decrease in platelet counts (*n* = 16, 80.0%). There were no treatment associated fatal cases with one case being discontinued from treatment due to AE. The median time to response (mTTR) was 2.1 months (95% CI: 2.0–2.1) while the median duration of response (mDOR) was 5.9 months (95% CI: 4.8–7.2). The PFS of sintilimab-chemotherapy was 7.5 months (95% CI: 6.2–9.4), while mOS was not met by May 1, 2019. ORR was 85% (95% CI: 62.1–96.8%). Notably, DCR achieved 100.0% (95% CI: 83.2–100.0%). The OS rates for 6 months and 12 months were 100.0% and 68.0% respectively, suggesting a significant anti-tumor effect and benign tolerability. Interestingly, TMB was not associated with curative effects in the trial [25]. ATTRACTION-4 was a clinical trial of nivolumab combined with SOX/CapeOX among patients with advanced G/GEJ cancer as the first-line therapy. The ORR of nivolumab plus S-1 and oxaliplatin (SOX) was 57.1% (95% CI: 34.0–78.2), while that of nivolumab plus capecitabine and oxaliplatin (CapeOX) was 76.5% (95% CI: 50.1–93.2) [26]. Similarly, KEYNOTE-059 and KEYNOTE-062 determined the efficacy of pembrolizumab ± chemotherapy as the first-line therapy in advanced G/GEJ cancer [27, 28]. ORRs of the pembrolizumab group were 60.0% for KEYNOTE-059 [27] and 48.6% (CPS ≥ 1) for KEYNOTE-062 [28]. Comparatively, as a first-line option, the ORR of sintilimab with chemotherapy was superior to those of standardized chemotherapy, or pembrolizumab/nivolumab ± chemotherapy in advanced G/GEJ cancer.

### Cervical Cancer

For patients previously subjected to multiple chemotherapy sessions, recurrent or metastatic advanced cervical cancer (CC) (PD-L1 expression>1%, ECOG of 0–1), sintilimab (200 mg, intravenously, Q3W) combined with anlotinib (10 mg orally, QD, d1–14; every 21 days) was performed until disease progression, death or intolerable toxicity (ChiCTR1900023015). The study set ORR as the primary endpoint, while OS, PFS, and DCR were determined as the secondary endpoints. The maximum ORR and DCR were 54.5% and 100%, respectively. The mPFS and mOS had not been met by then, while the original regimen was deemed to be tolerable given that the most common AEs were G1/2 [29]. Due to their affordability, both sintilimab and anlotinib from China, are potential drugs for use as a combination regimen for CC.

### Other types of tumors

As the second-line option after chemotherapy, CR was achieved after the application of 3 cycles of sintilimab (200 mg Q3W) on an advanced intrahepatic cholangiocarcinoma (ICC) patient with low microsatellite instability (MSI-L) and TMB. Sintilimab was discontinued due to the adrenal insufficiency attack after a total of 34 cycles of ICIs, which was relieved by hydrocortisone (20 mg/d) administration [30]. Furthermore, as a third-line strategy, a trial of sintilimab combined with tegafurgimeracil-oteracil potassium capsules was performed on an ICC case with multiple metastases. PR was achieved after 6 cycles of treatment, and the survival quality of the patient was enhanced significantly [31].

Apart from the common cancer types, sintilimab was involved in suppressing tumor progression on a KRAS positive patient with hepatoid adenocarcinoma (HAC), a sort of rare tumor secreting AFP with morphological similarities to HCC, and eventually achieved a 52-month OS [32].

Analysis of the above clinical trials (Table 2) and specific cases showed the potential of sintilimab as an equivalent to pembrolizumab and nivolumab.

**Table 2:** Completed Sintilimab-based Clinical Trials

Identifier	Tumor	Line	Drug	Phase	Number	ORR(%)	DCR(%)	PFS (m)	OS (m)
NCT03114683 (ORIENT-1) [6]	Classical Hodgkin’s Lymphoma	≥ 2nd Lines	IBI308	II	96	80.4	97.8	NR	NR
NCT02937116 [25]	gastric/gastroesophageal junction adenocarcinoma	First Line	IBI308 + Capecitabine + oxaliplatin	Ib	20	85	100	7.5	NR
NCT02937116 [16]	non-squamous NSCLC	First Line	IBI308&PC	Ib	21	68.4	84.3	12.6	18.9
	squamous NSCLC	First Line	IBI308&GC	Ib	20	64.7	100	6.5	15.4
NCT03607539 (ORIENT 11) [14]	non-squamous NSCLC	First Line	IBI308 + Pemetrexed+ Platinum	III	378	51.9 VS 29.8	86.8 VS 75.6	8.9 VS 5.0	NR
NCT03629925 (ORIENT 12) [15]	squamous NSCLC	First-Line	IBI308 + GP	III	357	–	–	5.9	NR
NCT03628521 [13]	Advanced NSCLC	First-Line	IBI308 + anlotinib	I	22	77.3	100	NR	NR
ChiCTR-OIC-17013726 [12]	Resectable NSCLC (stage IA-IIIB)	Neoadjuvant	IBI308	Ib	30	20	90	NR	NR
ChiCTR1900023015 [29]	recurrent /metastatic advanced cervical cancer	≥ 2nd Lines	IBI308 + anlotinib	II	16	54.5	100	NR	NR
NCT03794440 (ORIENT-32) [24]	advanced HCC	First-line	IBI308+ IBI305	II	24	25	83.3	8.4	NR

*NSCLC* non-small cell lung cancer, *NR* not reached, *P* pemetrexed, *C* cisplatin, *G* gemcitabine, *PC* pemetrexed + cisplatin, *m* month

## Adverse effects

Not only effectiveness but also safety is of concern when using sintilimab in solid tumors. ICIs activate the immune system in patients with cancer and promote the in vivo killing effect of T cells. Excessive activation of the immune-system leads to immune-related adverse events (irAEs) in the human body [33]. Primary causes of irAEs mechanism are the disturbance of immune balance, which might be explained by several potential hypotheses. For instances, when immune cells are over-active, cytotoxic T cells cross-react between tumor and normal cells. In addition, the increased inflammatory factors as well as the rise in the number of auto-immune antibodies, explain this phenomenon [34–36]. The irAEs are characterized by diarrhea, hepatitis, rash, pneumonia, thyroid dysfunction and others [33].

Common sintilimab-associated irAEs are characterized by pyrexia, hypothyroidism, rash, pneumonitis, fatigue and decreased platelet count [6, 12, 25]. Infrequent irAEs symptoms include PNSs [21], immune induced-myositis/myocarditis and rhabdomyolysis [37], high-CPK asymptomatic hypothyroid myopathy [38], cardiac toxicity [18], and adrenal insufficiency [30]. Furthermore, a refractory colon cancer patient exhibited systemic inflammatory response syndrome (SIRS) after sintilimab administration, leading to pulmonary fibrosis [39]. Cytokine release syndrome (CRS) along with multiple-organ injury was incurred after several cycles of sintilimab combined with chemotherapy on a patient with unresectable esophageal cancer (ESC) [40]. However, the above irAEs are controllable, and consistent with known toxicities.

Anti-PD-1/PD-L1 blockades presented a high synergistic effect when incorporated with other treatments [41, 42]. Previous studies revealed the anti-angiogenesis drugs potential to improve the efficacy of immunotherapy by reducing tumor hypoxia, improving perfusion, and reforming the tumor microenvironment [43, 44]. For example, clinical trials on the treatment of advanced HCC using immune checkpoint inhibitors alone exhibited a poor efficacy [45, 46]. However, immunotherapy combined with anti-angiogenesis therapy presented a synergistic effect on the mechanism of action [23, 46], which was proved by a trial of IMbrave 150 in unresectable HCC patients [23]. Strategies that combine other types of immunomodulators, chemotherapy, and molecular targeted treatments either in vivo or in vitro are ongoing [5].

Over the past few decades, clinical trials of new drugs in China were lagging behind other regions and their approval in the Chinese market were delayed for years. With the advance of the National Medicine Products Administration (NMPA) reform, more international pharmaceutical companies will perform clinical trials in China after realizing the globalization of drug development [47]. Interestingly, sintilimab can potentially benefit patients from underdeveloped areas or developing countries with promising anti-tumor effects as well as its affordability.

Several sintilimab-based clinical trials on malignancies are ongoing in multi-center in China, among the key trials are elaborated in (www.clinicaltrials.gov/)(Table 3):

**Table 3:** Main Ongoing Clinical Trials

Title	Identifier	Phase	Line	Intervention	Sample size	Primary ending points
HCC						
TACE Combined with Anti-PD-1 Antibody in Patients with Advanced Hepatocellular Carcinoma	NCT04297280	II	1st Line	IBI308 plus TACE	25	ORR
Neoadjuvant Therapy for Hepatocellular Carcinoma	NCT04174781	II	Neoadjuvant	IBI308 plus TACE	61	PFS
Ib Study of the Efficacy and Safety of IBI310 Combined with Sintilimab in Advanced Hepatocellular Carcinoma	NCT04401813	Ib	2nd Line	IBI308 plus IBI310	47	AE&ORR
A Study to Evaluate the Efficacy and Safety of Sintilimab in Combination with IBI305 (Anti-VEGFR Monoclonal Antibody) Compared to Sorafenib as The First-Line Treatment for Advanced Hepatocellular Carcinoma	NCT03794440	II/III	1st Line	IBI308 plus IBI305 VS sorafenib	566	OS& PFS
Combination of Sintilimab and Stereotactic Body Radiotherapy in Hepatocellular Carcinoma (ISBRT01)	NCT04167293	II/III	2nd Line	SBRT plus IBI308 VS SBRT	116	PFS
Anlotinib Hydrochloride Capsules Combined with Sintilimab Injection in the Treatment of Advanced Hepatocellular Carcinoma (HCC)	NCT04052152	II	1st Line	Anlotinib plus IBI308	20	ORR & ARR
A Study of Sintilimab Combined with Apatinib and Capecitabine in Advanced Hepatocellular Carcinoma	NCT04411706	II	1st Line	IBI308 plus apatinib plus capecitabine	46	ORR
Safety and Efficacy of Radiotherapy Plus Sintilimab for HCC with Portal Vein Tumor Thrombosis	NCT04104074	I	NA	IBI308 plus radiotherapy	20	AEs
Safety and Efficacy Study of Sintilimab Combined with IBI305 in Patients with Advanced Hepatocellular Carcinoma	NCT04072679	I	1st Line	IBI308 plus IBI305	45	AEs
Microwave Locoregional Treatment with Immunotherapy for Unresectable HCC	NCT04220944	I	1st Line	IBI308 plus TACE plus Microwave Ablation	45	PFS
TAI Combined with PD-1 Inhibitor in Locally Advanced, Potentially Resectable HCC	NCT03869034	II	1st Line	TAI(FOLFOX) plus IBI308	40	PFS
Pediatric Tumors						
Sintilimab in the Treatment of Advanced and Refractory Pediatric Malignant Tumors	NCT04400851	I	NA	IBI308	18	MTD
NSCLC						
Sintilimab Combined with Bevacizumab for Brain Metastases from Non-small Cell Lung Cancer	NCT04213170	II	1st Line	IBI308 plus Bevacizumab	60	iORR
Anlotinib Plus Sintilimab for NSCLC Patients with First-generation EGFR-TKIs Drug Resistance Along with T790M Negative	NCT03765775	II	NA	IBI308 plus anlotinib	20	PFS
Sintilimab Combined with Docetaxel for Standard Chemotherapy Failure Non-driver Gene Mutation Metastatic Non-small Cell Lung Cancer	NCT04144582	II	2nd Line	IBI308 plus Docetaxel	30	ORR
Consolidation Sintilimab After Concurrent Chemoradiation in Patients with Unresectable Stage III NSCLC	NCT03884192	II	1st Line	IBI308 plus Docetaxel	30	ORR
Safety and Tolerability Evaluation of Sintilimab in Combination with Radiation in Stage IV NSCLC Patients	NCT03812549	I	1st Line	SBRT plus IBI308	29	AEs
Efficacy and Safety of Sintilimab With or Without Radiotherapy in Patients with Recurrent or IV NSCLC (EGFR -, ALK -) After Failure of Platinum-based Chemotherapy: A Randomized, Open Labled, Phase II Clinical Study	NCT04513301	II	NA	IBI308 alone or IBI308 plus Radiotherapy	70	ORR & AEs
SCLC						
Assessing Safety and Efficacy of Sintilimab and Metformin Combination Therapy in SCLC	NCT03994744	II	NA	IBI308 plus Metformin	68	ORR & Safety
Anlotinib Combined with Sintilimab as Second-line Treatment or Beyond in Patients With Small Cell Lung Cancer	NCT04192682	II/III	2nd Line	IBI308 plus anlotinib	40	PFS
The Efficacy of PD-1 Antibody Sintilimab on Early-stage Multiple Primary Lung Cancer With Ground Glass Density.	NCT04026841	II	1st Line	IBI308	36	ORR
Colorectal Cancer						
Anlotinib Plus Sintilimab as First-line Treatment for Patients with Advanced Colorectal Cancer (APICAL-CR)	NCT04271813	II	1st Line	IBI308 plus anlotinib	30	ORR
Phase II Study to Evaluate the Efficacy and Safety of Fruquintinib Plus Sintilimab as Third-line Therapy for Colorectal Cancer	NCT04179084	II	3rd Line	IBI308 plus fruquintinib	30	ORR
IBI310 in Combination with Sintilimab in Patients with DNA Mismatch Repair Deficient(dMMR)/Microsatellite Instability High (MSI-H) Locally-advanced or Metastatic Colorectal Cancer	NCT04258111	II	NA	IBI308 plus IBI310	68	ORR
Esophageal Cancer						
Sintilimab in Combination with Chemotherapy in Neoadjuvant Treatment of Potentially Resectable Esophageal Cancer	NCT03946969	I/II	Neoadjuvant	IBI308 plus Chemo	40	AEs
Sintilimab Plus Chemotherapy Followed by dCRT in Locally Advanced ESCC	NCT03985046	I	NA	IBI308 plus Chemo	25	LCR
Gastric Cancer						
Efficacy and Safety Evaluation of Sintilimab or Placebo in Combination with XELOX as First Line Treatment in Patients with Gastric Cancer	NCT03745170	III	1st Line	IBI308 or Placebo plus Chemo	650	OS
The Purpose of This Study is to Evaluate the Efficacy and Safety of Sintilimab in Combination with Xelox as Neoadjuvant Therapy for Patients with Resectable Locally Advanced Gastric or Gastroesophageal Adenocarcinoma.	NCT04065282	II	Neoadjuvant	IBI308 plus Xelox	36	pCR
Cervical Cancer						
Combination of PD-1 Monoclonal Antibody and HPV Vaccine in Patients with Cervical Cancer	NCT04096911	II	2nd Line	IBI308 plus quadrivalent HPV vaccine	20	ORR
Ovarian Cancer						
An Open-label, Phase I/II Study of the Pan-immunotherapy in Patients with Relapsed/Refractory Ovarian Cancer	NCT03989336	I/II	≥3rd Line	IBI308 plus Chemo plus Manganese Chloride	80	ORR & AEs
ICC						
Systemic Chemotherapy Plus PD-1 for Metastasis ICC	NCT04398927	II	1st Line	IBI308 plus FOLFIRI	25	PFSR
Biliary Tract Cancer						
Anlotinib in Combination with PD1 With Gemcitabine Plus (+) Cisplatin for Unresectable or Metastatic Biliary Tract Cancer	NCT04300959	II	1st Line	IBI308 plus anlotinib plus Chemo	80	12 m-OS rate
Lymphoma						
Sintilimab with P-GemOx Regimen for Newly Diagnosed Advanced Extranodal Natural Killer/T-cell Lymphoma, Nasal Type	NCT04127227	II	1st Line	IBI308 plus pegaspargase plus chemo	63	ORR & complete remission rate
Sintilimab (IBI308) in Refractory or Relapsed PMBCL, PT/NKCL and PCNSL	NCT04052659	II	≥3rd Line	IBI308	30	ORR
Nasopharyngeal Carcinoma						
Sintilimab (PD-1 Antibody) and Chemoradiotherapy in Locoregionally-advanced Nasopharyngeal Carcinoma	NCT03700476	III	NA	IBI308 plus Chemo	420	FFS
Soft Tissue Sarcoma						
The Combination of Sintilimab and AI (Doxorubicin, ADM/Ifosfamide, IFO) for the First Line Treatment of Select Type of Metastatic/Unresectable Soft Tissue Sarcoma	NCT04356872	II	1st Line	IBI308 plus Doxorubicin Hydrochloride plus Ifosfamide	45	ORR

*TACE* transarterial chemoembolization, *ORR* objective response rate, *iORR* intracranial objective response rate, *OS* overall survival, *PFS* progression-free survival, *PFSR* progression free survival rate, *AE* adverse event, *ARR* adverse reaction rate, *AER* incicende of adverse events, *TAI* transarterial infusion chemotherapy, *MTD* maximum tolerable dose, *SBRT* stereotactic body radiation therapy, *LCR* local control rate, *pCR* pathological complete response rate, *FFS* failure-free survival, *NA* not applicable

## Conclusions

This article summarizes the progress of sintilimab in the field of cancer treatments, generalizing the efficacy and safety of sintilimab alone or combined regimens in multiple solid tumors. With the promising potential and application prospect of sintilimab, a vast number of multicenter, large-scale prospective clinical trials are going ahead in China to further determine its safety and effectiveness for cancer therapy. Hitherto, sintilimab has only been approved for use in RR-cHL, and it could be expected to be approved for more cancer types and admitted to realize its globalization.

## Data Availability

All data analysed during this study are included in this published article.

## Abbreviations

PD-1: Programmed cell death protein 1
PD-L1/2: Programmed cell death protein 1 ligand 1/2
CTLA-4: Cytotoxic T lymphocyte antigen-4
ICIs: Immune checkpoint inhibitors
ORR: Objective response rate
DLBCL: Diffuse large B cell lymphoma
NSCLC: Non-small lung cancer
MPR: Major pathologic response
pCR: Pathologic complete responses
PET-CT: Positron emission tomography-computed tomography
TMB: Tumor mutation burden
DCR: Disease control rate
EGFR: Epidermal growth factor receptor
ALK: Anaplastic lymphoma kinase
PFS: Progression-free survival
OS: Overall survival
TTR: Time to response
DOR: Duration of response
ESMO: European Society for Medical Oncology
PR: Partial remission
TPS: Tumor proportion score
mTTP: median time to tumor progression
CR: Complete remission
AFP: Alpha-fetoprotein
HCC: Hepatocellular carcinoma
CC: Cervical cancer
ICC: Intrahepatic cholangiocarcinoma
HAC: Hepatoid adenocarcinoma
G/GEJ: Gastric/gastroesophageal junction adenocarcinoma
WBC: White blood cell
TRAE: Treatment related adverse event
